# Orthotopic and metastatic tumour models in preclinical cancer research

**DOI:** 10.1016/j.pharmthera.2024.108631

**Published:** 2024-05

**Authors:** Stephen M. Stribbling, Callum Beach, Anderson J. Ryan

**Affiliations:** a Department of Chemistry, University College London, Gower Street, London WC1E 6BT, UK; b Department of Oncology, University of Oxford, ORCRB, Roosevelt Drive, Oxford OX3 7DQ, UK; c Fast Biopharma, Aston Rowant, Oxfordshire, OX49 5SW, UK

**Keywords:** Preclinical, Drugs, Orthotopic, Metastatic, Xenograft, Syngeneic, PDX, Organoids

## Abstract

Mouse models of disease play a pivotal role at all stages of cancer drug development. Cell-line derived subcutaneous tumour models are predominant in early drug discovery, but there is growing recognition of the importance of the more complex orthotopic and metastatic tumour models for understanding both target biology in the correct tissue context, and the impact of the tumour microenvironment and the immune system in responses to treatment. The aim of this review is to highlight the value that orthotopic and metastatic models bring to the study of tumour biology and drug development while pointing out those models that are most likely to be encountered in the literature. Important developments in orthotopic models, such as the increasing use of early passage patient material (PDXs, organoids) and humanised mouse models are discussed, as these approaches have the potential to increase the predictive value of preclinical studies, and ultimately improve the success rate of anticancer drugs in clinical trials.

## 1 Introduction

While significant progress has been made in cancer treatment over recent decades (Howlader et al., 2021; Scott et al., 2023), novel approaches to the development of new drugs remains crucial to address continuing unmet clinical needs (Cui et al., 2020; Kunnumakkara et al., 2019; Schlander, Hernandez-Villafuerte, Cheng, Mestre-Ferrandiz, & Baumann, 2021; Sonawane, Wagh, Dhumane, & Deore, 2019). Several major (Belluomini et al., 2022; Bupathi, Kaseb, Meric-Bernstam, & Naing, 2015; Krishnan, 2023; Moss, Beal, & Tabar, 2022) and rare cancer types (Wang et al., 2023) have limited or no effective treatment options for advanced disease; patients develop resistance to existing treatments (Cree & Charlton, 2017; Nussinov, Tsai, & Jang, 2021) leading to treatment failure and disease progression, and drugs that target metastatic processes, such as tumour cell migration, invasion, and colonization, are needed to prevent and treat metastatic disease (Gomez-Cuadrado, Tracey, Ma, Qian, & Brunton, 2017).

In vivo tumour models provide a critical bridge between preclinical research and clinical trials in anti-cancer drug development (Fiebig, Maier, & Burger, 2004; Ireson, Alavijeh, Palmer, Fowler, & Jones, 2019; Ruggeri, Camp, & Miknyoczki, 2014; Sausville & Burger, 2006; Teicher, 2005; Teicher, 2006; Teicher, 2009; Workman et al., 2010). These models are used to evaluate drug pharmacology, pharmacokinetics, pharmacodynamics, efficacy, toxicity, and safety profiles, as well as facilitating biomarker development and patient selection strategies (Teicher, 2005; Teicher, 2006; Teicher, 2009). In vivo tumour models also play an essential role in lead optimization before candidate drugs are selected and advanced into GLP toxicology studies prior to human trials (Ireson et al., 2019; Teicher, 2005; Teicher, 2006; Teicher, 2009).

Subcutaneous tumours are the most widely used in vivo model in preclinical cancer research because of the practical advantages they offer (Gengenbacher, Singhal, & Augustin, 2017). Subcutaneous tumours are generally straightforward to establish and monitor, and they grow rapidly and reliably, making them convenient models for screening the effects of new molecules on tumour growth, regression, or response to treatment thereby providing valuable data to guide the future design and prioritization of potential drug candidates (Morton & Houghton, 2007; Stribbling & Ryan, 2022). Nonetheless, subcutaneous tumours lack the complex microenvironment and interactions found in the original organ/tissue, potentially limiting the translatability of results to clinical settings (Gengenbacher et al., 2017). Therefore, additional models, such as orthotopic and metastatic models, are employed to complement the findings from subcutaneous models and provide a better understanding of cancer biology in response to therapy.

Orthotopic and metastatic tumour models allow drugs to be evaluated in more clinically relevant biological contexts. By considering target expression, drug distribution and the interactions between tumour cells, non-tumour stromal components, blood vessels and the host immune system, these models provide a more comprehensive assessment of treatment responses. Data generated in orthotopic models may more closely resemble the clinical disease and may be a more accurate guide to biomarker development and decision-making in clinical trials thereby improving the predictive value of preclinical findings (Antonello & Nucera, 2014; Bibby, 2004; Cook, Jodrell, & Tuveson, 2012). However, the use of orthotopic and metastatic tumour models for drug evaluation poses some practical difficulties. For example, the surgical procedures, post-operative care, and monitoring of tumour growth at specific anatomical sites all add complexity and can increase overall experimental timelines (Cook et al., 2012). Additionally, orthotopic tumour models generally have higher experimental variability compared to subcutaneous models (Flatmark, Maelandsmo, Martinsen, Rasmussen, & Fodstad, 2004; Lv et al., 2020) meaning larger group sizes may be required to reliably assess drug effects.

The aim of this review is to review the use of orthotopic and metastatic tumour models in preclinical cancer research, outlining some of their main advantages and limitations, and highlighting considerations associated with the use of orthotopic and metastatic tumour models as part of anti-cancer drug development.

## 2 Orthotopic tumour models

Orthotopic tumour models aim to replicate the clinical setting of tumour growth and progression by implanting tumour cells or tissues at their anatomically correct or appropriate location within the body of an experimental animal (Bibby, 2004; Hiroshima et al., 2016; Killion, Radinsky, & Fidler, 1998a; Talmadge, Singh, Fidler, & Raz, 2007). The term “orthotopic” comes from the Greek words “*ortho*,” meaning correct or proper, and “*topos*,” meaning pertaining to a place, and it can be defined as “*located in the proper anatomical position”*. A goal of orthotopic tumour models is to recreate the natural microenvironment of the tumour site, including tissue-specific architecture, cell-cell interactions, and vasculature, to better mimic the complex interactions and behaviour of tumours in the human body (Bibby, 2004; Hiroshima et al., 2016; Killion et al., 1998a).

Unlike subcutaneous models, which involve the ectopic (“*out of place*” or “*abnormal place or position*”) implantation of tumour cells or tissue fragments beneath the skin, orthotopic models reproduce the primary tumour location by, for example, injecting cells or fragments of glioblastoma (Joo et al., 2013; Zhao et al., 2012), prostate cancer (Cifuentes, Valenzuela, Contreras, & Castellon, 2015; Hughes, Simons, & Hurley, 2017; Saar et al., 2015; Wang et al., 2005) or non-small cell lung cancer (Justilien & Fields, 2013; Shibuya et al., 2007; Takahashi et al., 2012a; Wang, Fu, Kubota, & Hoffman, 1992) into the brain, prostate or lung, respectively.

Many anatomical sites have been used to set up orthotopic tumour models in mice reflecting the wide variety of human cancers being studied. As well as the four most commonly diagnosed cancers – breast, prostate, lung and colon (Cifuentes et al., 2015; Fu, Besterman, Monosov, & Hoffman, 1991; Fu, Le, & Hoffman, 1993; Okano et al., 2020; Saar et al., 2015; Wang et al., 2005; Wang, Fu, Kubota, & Hoffman, 1992) - there are significant proportions of patients with newly diagnosed cancers at other anatomical sites including pancreas, bladder, ovary and liver, and each anatomical site represents an important disease for orthotopic cancer models (Decio & Giavazzi, 2016; Erstad et al., 2018; Fu, Guadagni, & Hoffman, 1992; Fu & Hoffman, 1993; Naito, Higuchi, Shimada, & Kakinuma, 2020; Wang, Luan, Goz, Burakoff, & Hiotis, 2011).

Human tumour cell-line derived xenografts (CDXs) (from “*xeno-”* foreign; graft from one species to an unlike species) are the most widely used subcutaneous tumour model (Gengenbacher et al., 2017; Oliveira, Abrantes, Tralhao, & Botelho, 2020) and they are also a widely used orthotopic tumour model system (Table 1a). For xenograft models such as these, tumour cells/fragments are injected/implanted into immune-deficient mice, many strains of which are now available (Table 2) (Chulpanova, Kitaeva, Rutland, Rizvanov, & Solovyeva, 2020; Olson, Li, Lin, Liu, & Patnaik, 2018; Puchalapalli et al., 2016; Stribbling & Ryan, 2022). In contrast, murine tumour cell lines (Table 1b) can be grown in genetically matched (syngeneic) fully immune-competent mice, using widely available inbred strains such as C57BL/6 and BALB/c (Chulpanova et al., 2020; Li, Feuer, Ouyang, & An, 2017; Nolan et al., 2020; Potter, 1985).

**Table 1:** Examples of human xenograft and mouse syngeneic orthotopic tumour models.

(a) Human cell line derived orthotopic xenograft tumour models			
Cancer type	Cell line	Injection site	Refs.
Breast	T-47D	mammary fat pad (s.c.)	Abu Quora et al. (2021)
	BT-549	mammary fat pad (s.c.)	Abu Quora et al. (2021)
	MDA-MB-231-mCherry	lactiferous duct	Malin, Chen, Schiller, Koblinski, and Cryns (2011)
Prostate	PC-3luc	dorsal lobe	McGovern et al. (2021)
	LNCaP-luc	dorsal lobe	McGovern et al. (2021)
	PC-3	right anterior lobe	Cifuentes et al. (2015)
	LNCaP	left or right dorsal lobe	Liu, Zhu, Ye, Zhu, and Wang (2022)
Lung	Calu-6-luc	left lateral thorax	Willoughby et al. (2020)
	H1299-GFP-luc	middle/upper lobe, right lung	Sosa Iglesias et al. (2019)
	A549-luc	left lung	Mordant et al. (2011)
	H441	left lung	Wu et al. (2007)
	H460	left lung	Takahashi et al. (2012b)
Colon	HCT-116-luc	caecal wall	Ravoori et al. (2019)
	HT-29	caecal wall	Georges et al. (2019)
	RKO	caecal wall	Georges et al. (2019)
Pancreas	Panc-1	tail of pancreas	Chen et al. (2022a)
	MIAPaca-2	pancreas	Huynh et al. (2011)
	L3.6pl	pancreas	Kleespies et al. (2005)
Ovarian	SKOV3-luc	ovarian bursa	Guo et al. (2017)
	IGROV-1	under ovarian bursa	Decio and Giavazzi (2016)
Stomach	TMK-1	wall of mid-stomach	McCarty et al. (2004)
	AGS-GFP-luc	serous side of stomach	Busuttil et al. (2018)
Liver	Huh7-Luci	median lobe surface	Qiu et al. (2021)
	PL5-luc	left hepatic lobe	Lu et al. (2007)
Kidney	Caki-2	left renal capsule	Linxweiler et al. (2017)
	786-O-luc	kidney	Cho et al. (2016)
Head & Neck	UM-SCC-1	floor of mouth	Simon et al. (1998)
	MDA1986	tongue	Myers, Holsinger, Jasser, Bekele, and Fidler (2002)
	ACC3, ACCM	parotid gland	Choi et al. (2008)
Oesophagus	TE-4	wall of oesophagus	Ohara et al. (2010)
	TE-8-luc	abdominal oesophagus	Kuroda et al. (2014)
Skin (melanoma)	A375	intradermal	Rozenberg, Monahan, Torrice, Bear, and Sharpless (2010)
Brain	U-87-MG	cortex/striatum junction	Bianco et al. (2017)
	U-87 MG	right brain	Sun et al. (2020)
Mesothelioma	EHMES-1, −10	thoracic cavity	Ogino et al. (2008)
Colon (liver metastases)	LS174T	intrasplenic	Kalber, Waterton, Griffiths, Ryan, and Robinson (2008)


(b) Murine cell line derived orthotopic syngeneic tumour models			
Cancer type	Cell line injected	Injection site	Refs.
Breast	4 T1	second mammary fat pad	Carrillo et al. (2023)
	4 T1-luc	fifth left breast	Dos Santos et al. (2018)
	EMT6-luc	mammary fat pad	Piranlioglu et al. (2019)
	EMT6	mammary fat pad	Amini et al. (2019)
Prostate	TRAMP-C2-luc	prostate	Lardizabal, Ding, Delwar, Rennie, and Jia (2018)
	Myc-CaP	anterior prostate lobe	Anker, Mok, Naseem, Thumbikat, and Abdulkadir (2018); Hughes et al. (2017)
Lung	LLC1-luc	left lung	Liu, Zhao, Senovilla, Kepp, and Kroemer (2021)
	LLC1	left lung	Hung et al. (2020)
	KLN205	lung parenchyma	Porrello et al. (2018)
Colon	MC38	caecal wall	Greenlee and King (2022)
	MC38-luc	rectal wall	Uccello et al. (2022)
	CT-26	caecal wall	Kruse et al. (2013)
	CT26-luc	caecum	Evans et al. (2019)
Bladder	MB49	bladder wall	Cai et al. (2022)
	MBT-2	intravesical space	Chan et al. (2009)
Pancreas	Panc02	head of pancreas	Partecke et al. (2011)
	Pan02-CAG-luc2	tail of pancreas	Luheshi et al. (2016)
Ovary	ID8-F3mCherryluc	intra-peritoneal	Gonzalez-Pastor et al. (2019)
	ID-8 GFP-luc	intra-bursal	Lin, Sun, Wu, and Wang (2017)
Liver	Hepa1–6	beneath Glissons capsule	Wang et al. (2011)
	Hepa1–6 GFP	portal vein	Limani et al. (2016)
	TIB-75 GFP	portal vein	Limani et al. (2016)
Kidney	Renca	renal subcapsule	Matin et al. (2010)
	Renca-luc	kidney parenchyma/capsule	Ding, Wang, and Chang (2018)
Skin (melanoma)	B16F10	intra-dermal (flank)	Rossi et al. (2019)
	B16F10	intra-dermal (left pinna)	Fowlkes et al. (2019)
Brain	GL261	right cerebral hemisphere	Lumniczky et al. (2002)
	GL261-GFP-Fluc	right hemisphere	Khalsa et al. (2020)


(c) Human patient-derived orthotopic xenograft (PDOX) tumour models			
Cancer type	Material engrafted	Engraftment site	Refs.
Breast	tumour fragments	mammary fat pad (s.c.)	Sommaggio et al. (2020)
	tumour fragments	mammary fat pad (s.c.)	Okano et al. (2020)
	tumour cell suspension	intra-ductal	Behbod et al. (2009)
	tumour fragments	mammary fat pad (s.c.)	Fu et al. (1993)
Prostate	tumour fragments	sub-renal capsule	Salem et al. (2020)
	tumour fragments	sub-renal capsule	Wang et al. (2005)
	tumour fragments	dorsal prostate lobe	Saar et al. (2015)
Colon	tumour cell suspension	wall of colon	Puig et al. (2013)
	tumour cell suspension	wall of colon	De Angelis et al. (2022)
	tumour fragments	surface of intestine	Fu et al. (1991)
Lung	tumour fragments	left lung	Wang, Fu, and Hoffman (1992)
	tumour fragments	parietal pleura	Astoul, Wang, Colt, Boutin, and Hoffman (1996)
Ovary	tumour fragments	beneath ovarian capsule	Fu and Hoffman (1993)
Pancreas	tumour fragments	surface of pancreas	Fu et al. (1992)
Stomach	tumour fragments	stomach serosa	Furukawa, Kubota, Watanabe, Kitajima, and Hoffman (1993)

**Table 2:** Immune-deficient mouse strains commonly used for orthotopic and experimental metastasis models (Adigbli et al., 2020; Chen et al., 2022b; Nagatani et al., 2019; Shultz et al., 2014).

Name	Strain	B cells	T cells	Dendritic cells	Macrophages	NK cells	Complement	Spontaneous tumours	Leaky
Nude	*Foxn1*^*nu*^	Yes	No	Yes	Yes	Yes	Yes	No	No
SCID	*Prkdc*^*scid*^	No	No	Yes	Yes	Yes	Yes	No	Yes
NOD SCID	NOD. *Prkdc*^*scid*^	No	No	Yes	Defective	Defective	Defective	Yes	Yes
NSG	NOD. *Prkdc*^*scid*^*Il2rg*^*tm1Wjl*^	No	No	Defective	Defective	No	Defective	No	No
NOG⁎	NOD. *Prkdc*^*scid*^*Il2rg*^*tm1Sug*^	No	No	Defective	Defective	No	Defective	No	No

⁎ NOG mice have an *Il2rg* mutation producing a protein that will bind cytokines but not signal. NSG mice have a null mutation whereby no Il2rg is expressed and cytokines cannot bind.

Syngeneic and CDX tumour models are usually set up using immortalised tumour cell lines that have been sub-cultured/passaged over many years either in vitro (in growth-factor rich tissue culture medium), grown adhered to tissue culture plates and dissociated with trypsin/EDTA or alternatively in vivo, as subcutaneous tumours, which are excised and cut into fragments or disaggregated before re-implantation. These culturing methods select for rapid tumour growth and survival (either on plastic or subcutaneously) and can cause substantial and irreversible changes to cell biology (Daniel et al., 2009; Gillet et al., 2011; Hausser & Brenner, 2005), such that these cell line models may have limited predictive value (Johnson et al., 2001).

The problem can be overcome to a certain extent by using patient-derived xenograft (PDX) models (Abdolahi et al., 2022; Hidalgo et al., 2014). Fresh tumour tissue derived from treatment-naïve primary or metastatic tumours is obtained during surgery or from biopsies (or occasionally, from ascites) (Abdolahi et al., 2022; Calles, Rubio-Viqueira, & Hidalgo, 2013; Kim et al., 2009; Whittle, Lewis, Lindeman, & Visvader, 2015). Typically, the tumour is cut into small (∼1–2 mm^3^) pieces or disaggregated enzymically or mechanically to give a cell suspension, and then engrafted or injected ectopically into immune-deficient mice, with a number of potential sites being available, including subcutaneously in the flank, in the anterior compartment of the eye, under the renal capsule, or into the intracapsular fat pad (Abdolahi et al., 2022). More recently, PDX models have also been established from patient-derived circulating tumour cells (CTCs) rather than the tumour itself (Ramani et al., 2019; Tayoun et al., 2019). PDX tumours can also be engrafted orthotopically into the same organ as the original tumour and grown as patient-derived orthotopic xenografts (PDOXs). PDX and PDOX tumours maintain much of the structure and composition of the parent tumour and more accurately recapitulate the human disease (Day, Merlino, & Van Dyke, 2015; DeRose et al., 2011; Pompili, Porru, Caruso, Biroccio, & Leonetti, 2016; Rubio-Viqueira et al., 2006; Talmadge et al., 2007; Tentler et al., 2012; Zhao et al., 2012). As a result, the response of patients to certain types of therapy may be more accurately predicted (Garrido-Laguna et al., 2011; Pompili et al., 2016; Rosfjord, Lucas, Li, & Gerber, 2014; Talmadge et al., 2007). Importantly, PDOX models better mimic clinical metastases than subcutaneous PDX models, suggesting they have greater biological and clinical relevance (Hoffman, 2015). Examples of PDOX models set up using fresh human tumour tissue are given in Table 1c.

PDX tumours are routinely grown and expanded subcutaneously, where human stromal tissue initially present is replaced by mouse stroma over a small number of passages (Chao et al., 2017; Invrea et al., 2020), and later passages may favour genetic drift or tumour cells that grow and survive better in the subcutaneous setting of the mouse (Zhuo et al., 2020). Because of this, PDX and PDOX studies are often conducted using low passage tumours due to the possibility that each passage to a new host may dilute the features present in the original patient tumour (Chao et al., 2017; Rosfjord et al., 2014; Shi, Li, Jia, & Fan, 2020).

Orthotopic tumour models have both advantages and disadvantages (Bibby, 2004) (Table 3), with the site of tumour growth itself being something of a double-edged sword: it makes it possible to recapitulate and accurately model human disease; but it also makes model preparation and the subsequent determination of tumour growth and metastasis more difficult than with subcutaneous models. In addition, there are important ethical concerns when considering the use of orthotopic models. Because the implantation of cancer cells to create an orthotopic model is an invasive procedure, sometimes requiring complex surgery, researchers have to consider the overall well-being of the animals and ensure that procedures are performed with the utmost care to minimize discomfort, pain, and distress. Ethical guidelines typically require the use of anaesthesia, proper analgesia, and other measures to ensure humane treatment. For example, careful consideration is required to accurately determine both the extent and position of tumour growth so that animal welfare is not significantly negatively impacted during a study (Workman et al., 2010). Because of the potential welfare issues a strong justification for the use of orthotopic models based on the scientific necessity and relevance of the model to the research question is required in order to balance the potential benefits to human health or scientific knowledge and the potential harm to the animals. Most countries have established strict regulatory and reporting guidelines to ensure ethical and humane treatment of animals in research, for example the Animals (Scientific Procedures) Act 1996 [UK], the Animal Welfare Act [USA], and Directive 2010/63/EU on the Protection of Animals Used for Scientific Purposes [EU] (Workman et al., 2010).

**Table 3:** Advantages and disadvantages of orthotopic tumour models.

Advantages	Refs.
• Cell-derived and patient-derived tumours grow in their tissue of origin, preserving many biological characteristics of the original tumour. This results in a more accurate recapitulation of the human disease, enhancing the predictive value of the models and the clinical significance of the results obtained.	Bibby (2004); DeRose et al. (2011); Fiebig et al. (2004); Hiroshima et al. (2016); Manzotti, Audisio, and Pratesi (1993); Mattie et al. (2013); Ruggeri et al. (2014); Teicher (2006); Tentler et al. (2012); Walters et al. (2013); Waters, Janovitz, and Chan (1995); Whiteford et al. (2007); Zhao et al. (2012)
• A variety of human and murine tumour cell lines are available for orthotopic injection, and increasingly these have been stably transfected to enable the production of a bioluminescent signal.	ATCC (2023); ECACC (2023); PerkinElmer (2023); RIKEN (2023); Teicher (2006)
• Tumour cells stably expressing luciferase enables solid tumour growth, metastasis and any therapeutic response (s) to be measured by bioluminescence (BLI, a method of choice) in a high-throughput, non-invasive manner.	Alsawaftah, Farooq, Dhou, and Majdalawieh (2021); Edinger et al. (2002); Jenkins et al. (2003); Liu, Su, Lin, and Ronald (2021); Sekar and Paulmurugan (2014); Weissleder (2002)
• Some tumour cell lines will only form solid tumours in vivo if injected orthotopically.	Kung (2007); Stephenson et al. (1992)
• Different strains of immune-competent and immune-deficient mice are commercially available to support the growth of orthotopic tumours.	Charles_River (2023); INOTIV (2023); Teicher (2006); The_Jackson_Laboratory (2023)

Disadvantages	Refs.

• The establishment of orthotopic tumour models often requires surgery, which can be technically challenging, expensive and time-consuming; as a result, low numbers of mice may be used per study reducing the statistical power of the experiments.	Bibby (2004); Loi et al. (2011); Richmond and Su (2008); Ruggeri et al. (2014); Smith, Merritt, Barr, and Thorley-Lawson (2011); Teicher (2006)
• The majority of orthotopic models are established in immune-deficient mice, making any study of the role of the immune system in tumour biology or therapy very difficult.	Herter-Sprie, Kung, and Wong (2013); Talmadge et al. (2007)
• Orthotopic tumours (except skin and mammary fat pad tumours) are not visible to the naked eye. Expensive, specialised non-invasive imaging techniques such as BLI, PET, CT, MRI and US are required to follow tumour growth and metastasis.	Kaijzel, van der Pluijm, and Lowik (2007); Liu, Su, et al. (2021); Lyons (2005); Sahai (2007); Sekar and Paulmurugan (2014); Serkova et al. (2021); van der Horst, Buijs, and van der Pluijm (2015); Weissleder (2002)
• The complexity of orthotopic models increases animal welfare considerations; the accurate determination of humane experimental end-points is challenging; the take rate for PDOX models is variable; and, tumour latency can be as long as 12 months.	Bibby (2004); Pompili et al. (2016); Ruggeri et al. (2014); Workman et al. (2010)
• A fresh tumour specimen is required to establish a patient-derived orthotopic model; ethical review processes together with obtaining the appropriate patient consents and permissions for future work can be time-consuming.	UK_Research_and_Innovation_(UKRI) (2023)

Although haematological tumours (e.g. lymphoma (Michel, Rosario, Andrews, Goldenberg, & Mattes, 2005), leukemia (Griessinger et al., 2018), myeloma (Lwin, Edwards, & Silbermann, 2016)) can be grown ectopically in non-physiological locations (e.g. as subcutaneous tumour or ascites), they can also be grown orthotopically as syngeneic, CDX or PDX/PDOX models (Kohnken, Porcu, & Mishra, 2017). For PDX studies, a distinct advantage of haematological tumours is that the source material is more readily available and easier to access than for solid tumours. Multiple patient samples can be obtained throughout the course of disease to model disease progression, and engraftment can recapitulate orthotopic systemic/disseminated disease (by i.v. injection) or orthotopic primary site disease (by intra-tibial or intra-femoral injection) (Griessinger et al., 2018; Richter et al., 2022).

## 3 Tumour organoid models

An organoid can be defined as a 3D structure grown from stem cells and consisting of organ-specific cell types that self-organizes through cell sorting and spatially restricted lineage commitment (Clevers, 2016). There has been growing interest in extending the concept of tissue organoids to encompass the use of patient-derived tumour organoids to create in vivo models (Bleijs, van de Wetering, Clevers, & Drost, 2019). To establish organoids, tumour cells can be derived from patient biopsies, surgically resected tumours, PDX tumours, or genetically engineered mouse models (Yoshida, 2020). Tumour cells are embedded in an extracellular matrix or scaffold that provides a three-dimensional structure for growth. Specific growth factors and nutrients are added to support the growth and survival of tumour cells and, over time, the tumour cells in 3D culture self-organise into organoids, forming tissue-like structures mimicking the cellular heterogeneity seen in the original tumour with expression of cell-specific markers and the development of differentiation-associated properties such as secretory functions in glandular tumours (Clevers, 2016; Jin et al., 2018; Porter, Murray, & McLean, 2020; Weeber, Ooft, Dijkstra, & Voest, 2017; Zhou, Cong, & Cong, 2021). Patient-derived tumour organoids have been established for a broad range of tumour types and have several practical advantages over patient-derived xenografts including a higher establishment success rate, more rapid establishment and the potential to generate matched normal control tissue (Nagle, Plukker, Muijs, van Luijk, & Coppes, 2018). Cancer cells can be converted between organoid culture and xenografts with high efficiency (Wang et al., 2022a) with the result that patient derived organoids have certain benefits of both 2-D cultured cells (e.g. ease of growth, genetic manipulation, implantation success) and PDXs (e.g. orthotopic implantation and metastasis, disease-relevant tumour microenvironment/stroma) (Okazawa et al., 2018; Yoshida, 2020). Examples of the use of organoid-derived orthotopic models are shown in Table 4. Orthotopic implantation of tumours or tumour organoids is also finding an important role as an adjunct to genetically engineered mouse models where the technique provides better control of tumour position and growth than stochastic tumour development (Jackstadt et al., 2019). and also allows more simple and rapid genetic manipulation thereby curtailing the need for further in vivo model development (Fumagalli et al., 2017).

**Table 4:** Examples of organoid-derived orthotopic tumour models (Wang, Xiang, Zhang, & Wang, 2022).

Cancer type	Mouse strain	Injection site	Refs.
Breast	Nude	4^th^ mammary fat pad	Sachs et al. (2018)
PDAC	Nude	Tail region of the pancreas	Boj et al. (2015); Boj et al. (2016)
GBM	NSG	Right cerebral cortex	Hubert et al. (2016); Jacob et al. (2020)
Rectum	NSG	Rectum (endoluminal)	Ganesh et al. (2019)
CRC	NSG	Colon lamina propria (mucosal)	Roper et al. (2017)
Ovary	NSG	Ovary (bursa)	Kopper et al. (2019)
Bladder	NOG	Bladder wall (submucosal)	Lee et al. (2018)

Organoids have some potential limitations, such as decreased cell diversity and heterogeneity compared with PDXs (Long, Xie, Shen, & Wen, 2022), and lack of accepted standardized methods for culture/propagation (Zhou et al., 2023a) but the benefits of these cells for establishing orthotopic/metastatic models and the ongoing development of disease-specific organoid biobanks (Andreatta et al., 2020; Beshiri, Agarwal, Yin, & Kelly, 2023; Betge & Jackstadt, 2023; Chang, Wu, Harnod, & Ding, 2022; Hollins & Parry, 2016; Jin et al., 2018; Kretzschmar, 2021; Lee et al., 2018; Li, Liu, & Chen, 2022; Low et al., 2021; Meijer, 2021; Ren, Chen, Yang, Li, & Xu, 2022; Seidlitz & Stange, 2021; Weeber et al., 2017; Yang, Wang, Wang, Zhang, & Wang, 2021; Yu et al., 2022; Zhou et al., 2021) suggest that they will find increased use in the future across a broader range of tumour types, including rare cancers (Li et al., 2022). Importantly, it has been shown that orthotopically implanted, patient-derived organoids have the potential to recapitulate patient responses in the clinic (Vlachogiannis et al., 2018).

## 4 Humanised mice

Human CDX, PDX and organoid orthotopic models require the use of immune-deficient mice with strains that exhibit severe immune deficiencies such as the NOD-SCID-IL2R gamma null (NSG) strain which is most often used for engraftment of primary human samples (Table 2) (Bresnahan, Lindblad, Ruiz de Galarreta, & Lujambio, 2020; Ito et al., 2002; Puchalapalli et al., 2016; Zhou, Facciponte, Jin, Shen, & Lin, 2014). The lack of an intact immune system is a significant limitation of orthotopic models grown in immune-deficient mice, but more recently NSG mice in particular have been used to establish humanised mouse models, where the defective mouse immune system is replaced (“humanised”) with e.g. human peripheral blood mononuclear cells (PBMCs) or hematopoietic stem cells (HSCs) to recapitulate the human immune system. This approach has enabled the role of the immune system in cancer therapy to be investigated using xenografted human tumours rather than syngeneic mouse tumour models (Cogels et al., 2021; Theocharides, Rongvaux, Fritsch, Flavell, & Manz, 2016; Tian, Lyu, Yang, & Hu, 2020; Zitvogel, Pitt, Daillere, Smyth, & Kroemer, 2016). Although humanised mice have several advantages, there are several potential limitations: they do not fully recapitulate the human immune system (Chen, Liu, Liu, & Yang, 2023); the human-derived PBMCs or HSCs are not patient-matched to the tumour (Ma, Pilvankar, Wang, Giragossian, & Popel, 2021); and, development of graft-versus-host disease (Ehx et al., 2018; Poirier, Dilek, Mary, & Vanhove, 2012) due to the interaction of the humanised immune system with mouse tissues may occur. Approaches to address some of these shortcomings are being investigated. For example, an immune-deficient mouse model has been developed which expresses human HLA instead of mouse MHC where the immune-deficiency can be corrected by transferring functional HLA-matched PBMCs resulting in an immune-competent mouse with a more human-like immune system (Morillon 2nd, Sabzevari, Schlom, & Greiner, 2020; Zeng et al., 2017). However, the potential impact of a humanised immune system on orthotopic tumours has not been widely studied, although a humanised tumour microenvironment enhanced both prostate tumour growth and metastasis (McGovern et al., 2018; McGovern et al., 2021) suggesting that tumour biology and response to therapy could be significantly affected.

## 5 Metastatic models

Metastasis (*change, movement from one point to another*) is the spread of cancer cells from the primary site of disease to another part of the body and along with invasion represents a key hallmark and differentiating feature between benign and malignant tumour growth (Hanahan & Weinberg, 2000; Hanahan & Weinberg, 2011). The metastatic process is of great significance since the majority of cancer-related deaths are due to the establishment and growth of metastases rather than to the growth of the primary tumour itself (Dillekas, Rogers, & Straume, 2019; Mehlen & Puisieux, 2006; Riihimaki, Thomsen, Sundquist, Sundquist, & Hemminki, 2018).

Metastasis is a highly complex process that can be broken down into a series of discrete steps commonly referred to as the metastatic cascade. These have been described in detail elsewhere (Fares, Fares, Khachfe, Salhab, & Fares, 2020; Ganesh & Massague, 2021; Welch & Hurst, 2019) and are outlined in Table 5.

**Table 5:** Biological steps leading to tumour metastasis.

Step	Biological Process
1.	**Tumour initiation** (mutation, oncogene expression, loss of tumour suppressor genes)
2.	**Primary tumour formation and growth**
3.	**Local invasion** (metastatic cells from the primary tumour migrate into the basement membrane and penetrate the underlying stroma)
4.	**Intravasation** (detached metastatic cells enter the general circulation (lymph, blood vessels) as circulating tumour cells (CTCs))
5.	**Circulation** (CTCs transported away from the primary tumour to distant sites around the body)
6.	**Arrest** (CTCs arrest or adhere to vessel walls in distant favourable capillary beds; pre-metastatic niches)
7.	**Extravasation** (viable CTCs cells invade vessel walls and then into the new tissue site)
8.	**Initial growth** (the tumour cells grow, and establish a conducive microenvironment for the formation of micrometastases by stimulating processes essential for their survival such as angiogenesis)
9.	**Colonization** (secondary tumour growth; micrometastases grow into clinically detectable metastases)
10.	**Further metastasis** (metastasis can occur from the initial metastases: “metastases from metastases”)

The transport of tumour cells from the primary to distant sites occurs mainly via the circulatory and lymphatic systems (Sleeman, Nazarenko, & Thiele, 2011), in theory enabling viable circulating tumour cells (CTCs) to spread and colonise almost any tissue in the body. However, in practice, for many primary tumours, the potential anatomical sites of secondary tumour growth are more restricted. Predating our current understanding, Paget in 1889 suggested that the outcome of metastasis depends upon interactions between the tumour cells and the host tissue, with the metastatic tumour cell (“seed”) being able to grow into a secondary tumour only once it has reached a sustaining organ environment (“soil”) (Fidler, 2003; Paget, 1989). Today, we can think of the seed in terms of e.g. cancer stem cells, progenitor cells or initiating cells, whereas the soil encompasses specific stromal and microenvironmental factors that together constitute an amenable pre-metastatic niche (Langley & Fidler, 2007; Talmadge & Fidler, 2010). In consequence, the site of formation of metastases can depend on specific interactions between the CTCs and the prospective host growth site leading to specific patterns of metastatic spread for specific cancer types, with the main organ sites of metastasis for the commonest cancers being liver, lung, bone and brain (Table 6).

**Table 6:** Most common sites of metastases (in Patients with Metastatic Disease).

Primary site	Proportion (%) of patients with metastases at organ site⁎
Breast	Bone 55%, Liver 36%, Lung 30%, Brain 18%
Prostate	Bone 89%, Liver 10%, Lung 7%
Colon	Liver 69%, Lung 31%, Peritoneum 14%, Bone 8%
Lung	Brain 41%, Bone 34%, Liver 23%, Lung 11%
Kidney	Lung 55%, Bone 35%, Liver 22%, Brain 18%
Ovary	Peritoneum 62%, Liver 20%, Pleura 14%, Lung 13%
Pancreas	Liver 77%, Lung 17%, Peritoneum 15%
Bladder	Bone 40%, Lung 31%, Liver 30%
Stomach	Liver 51%, Peritoneum 26%, Lung 13%, Bone 10%
Oesophagus	Liver 50%, Lung 35%, Bone 19%, Brain 9%
Liver	Liver 66%, Lung 19%, Peritoneum 16%, Bone 10%
Melanoma	Brain 47%, Lung 40%, Liver 28%, Skin 19%, Bone 17%
All sites	Liver 38%, Lung 27%, Bone 22%, Brain 13%, Peritoneum 11%

Data recalculated from Riihimaki et al. (2018).

⁎ Only most common sites shown. Totals may exceed 100% due to multiple sites of metastases in some patients.

The way in which in vivo models can recapitulate the different elements of the metastatic cascade depends on how they are set up. Although implantation of tumour cells orthotopically into the tissue site of origin can recapitulate the majority of the biological steps of metastasis (Table 5), the process is difficult to study experimentally (Hoffman, 1999). As a consequence, studying the spread and growth of tumours at clinically relevant sites of metastasis (rather than at the site of the primary tumour) has required the development of specialised metastasis models. In vivo models of metastasis can broadly be divided into two types: experimental and spontaneous, which can recapitulate different steps of the metastatic cascade or the entire cascade itself. Experimental models of metastasis are set up by the direct injection of tumour cells into the general circulation and depending upon the injection site used, metastases will subsequently develop at specific anatomical locations. The main injection routes used are:

• Intravenous (i.v., lateral tail vein): tumour cells become trapped in the lung microvasculature to recapitulate the formation of lung metastases;
• Intra-caudal (tail artery): tumour cells form metastases in the bones of (primarily) the hind limbs;
• Intracardiac (i.c., left ventricle): by bypassing the lung microvasculature the tumour cells disseminate more widely, promoting the formation of bone and liver metastases;
• Carotid artery: this takes tumour cells directly to the brain and is used for the formation of brain metastases;
• Intra-splenic/portal vein/mesenteric vein: these routes deliver tumour cells to the liver, which is a primary site of metastasis for several solid tumours such as colon cancer;
• Intra-iliac artery: this artery supplies blood to the legs, pelvis and pelvic organs and is primarily used for the formation of bone metastases;
• Intra-peritoneal (i.p): this route is used for the local dissemination of ovarian cancer cells and is also used to recapitulate orthotopic tumour formation in the peritoneal cavity;
• Intra-tibial (i.t.): tumour cell injection into this bone can be used to study the effects of metastases on bone structure and growth, especially osteolysis.

The tail vein, tail artery and i.p. routes of injection have the advantage that they do not require the mouse to be anaesthetised or undergo any surgical procedure and are thus relatively straightforward to perform. Other routes are technically more demanding, requiring both anaesthesia and surgery.

Table 7 gives examples of different tumour cell types injected via the routes described above to establish different experimental models of metastasis. It should be noted that injection of the same tumour cell line by different routes can be used to set up models that, when taken together, recapitulate the metastatic spread seen in the human disease. For example, if MDA-MB-231 human breast tumour cells are injected into the tail vein (i.v), metastases form primarily in the lungs, whereas metastases are seen primarily in the bone and liver if the intra-caudal route is used, and in the brain if the carotid artery route is used (Table 7a).

**Table 7:** Examples of experimental and spontaneous in vivo models of metastasis.

(a) Experimental models					
Tumour	Cell line	Model	Route of injection	Main site(s) of metastasis	Refs.
Breast	MDA-MB-231	xenograft	i.v. (lateral tail vein)	lung	Minn et al. (2005)
Breast	4 T1	syngeneic	i.v. (lateral tail vein)	lung	Pillar, Polsky, Weissglas-Volkov, and Shomron (2018)
Breast	MDA-MB-231	xenograft	i.c. (left ventricle)	bone	Dunn et al. (2009)
Prostate	RM1	syngeneic	i.c. (left ventricle)	bone	Jung et al. (2013)
Ovarian	A2780	xenograft	i.p.	mesentery	Shaw, Senterman, Dawson, Crane, and Vanderhyden (2004)
Ovarian	various	xenograft	i.p.	peritoneal cavity	De Haven Brandon et al. (2020)
Melanoma	K1735	syngeneic	intra-carotid	brain	Zhang, Lowery, and Yu (2017)
Breast	MDA-MB-231	xenograft	intra-carotid	brain	Zhang et al. (2017)
Prostate	various	xeno/syn	intra-splenic	liver	Simons, Dalrymple, Rosen, Zheng, and Brennen (2020)
Colon	LoVo	xenograft	intra-splenic	liver	Kim et al. (2020)
Colon	HT29	xenograft	intra-splenic	liver	Lavilla-Alonso et al. (2011)
Colon	various	syngeneic	intra-portal	liver	Limani et al. (2016)
Colon	HT-29	xenograft	intra-portal	liver	Thalheimer et al. (2009)
Colon	SW-620	xenograft	intra-portal	liver	Thalheimer et al. (2009)
Breast	various	syngeneic	intra-portal	liver	Goddard, Fischer, and Schedin (2016)
Breast	various	xenograft	intra-iliac	bone	Yu et al. (2016)
Prostate	various	xenograft	intra-iliac	bone	Nunez-Olle, Guiu, and Gomis (2021)
Breast	MDA-MB-231	xenograft	intra-tibial	bone	Peramuhendige et al. (2018)
Prostate	VCaP	xenograft	intra-tibial	bone	Eswaraka et al. (2014)
Various	various	xeno/syn	intra-caudal	bone	Kuchimaru et al. (2018)
Breast	MCF7	xenograft	intra-caudal	bone	Han et al. (2020)


(b) Spontaneous models					
Tumour type	Cells or tissue	Model type	Site of engraftment	Main site (s) of metastases	Refs.
Breast	MDA-MB-231 cells	xenograft	MFP	lymph nodes, lungs	Sommaggio et al. (2020)
Breast	patient specimen	xenograft	MFP	lungs, lymph nodes	Sommaggio et al. (2020)
Breast	patient specimen	xenograft	MFP	lungs	Fu et al. (1993)
Prostate	tumour fragments	xenograft	prostate gland	lymph nodes, pancreas, testis, liver	Salem et al. (2020)
Prostate	patient specimen	xenograft	anterior prostate	lymph nodes, lungs, liver, bone, kidneys, spleen	Wang et al. (2005)
Colon	patient specimen	xenograft	caecum wall	liver, lung, abdominal cavity	Puig et al. (2013)
Colon	patient specimen	xenograft	colon wall	liver, lung	De Angelis et al. (2022)
Colon	patient specimen	xenograft	colocaecal	lymph nodes, liver, abdominal cavity	Fu et al. (1991)
Lung	patient sample	xenograft	left upper lung	left and right lung, lymph nodes, chest wall pericardium	Wang, Fu, and Hoffman (1992)
Lung	patient sample	xenograft	parietal pleura	pleural cavity, lymph nodes	Astoul et al. (1996)
Ovarian	patient sample	xenograft	under ovary capsule	parietal peritoneum, colon, omentum, ascites	Fu and Hoffman (1993)
Pancreatic	patient sample	xenograft	surface of pancreas	lymph nodes, liver diaphragm, adrenal glands, stomach, duodenum, abdominal wall	Fu et al. (1992)
Stomach	patient specimen	xenograft	stomach serosa	lymph nodes, liver, peritoneum	Furukawa et al. (1993)

Experimental models of metastasis have a significant disadvantage in that they do not recapitulate the initial steps of the metastatic cascade such as growth at the primary tumour site, tissue invasion and/or intravasation (Gomez-Cuadrado et al., 2017). Spontaneous models of metastasis can recapitulate the entire metastatic cascade. A primary tumour can be established at subcutaneous or orthotopic sites and although metastasis is common in many orthotopic tumour models it is only very rarely seen in subcutaneous models, with some notable examples being the murine B16 melanoma and Lewis lung carcinoma (LLC) cell lines (Bertram & Janik, 1980; Fidler, 1973; Fidler, 2006; Gomez-Cuadrado et al., 2017).

As was described above, tumours growing orthotopically interact with their tissue of origin and more accurately recapitulate the human disease which may affect the initial steps of the metastatic cascade, enhancing the formation of metastases (Fidler, 2006). In certain models, such as the mammary fat pad model, a primary tumour can be surgically resected and the survival of the mouse prolonged allowing time for CTCs derived from the primary to develop into established metastases (Munoz et al., 2006). Although orthotopic CDX models are more common, PDOX models are increasingly being used as models of metastasis as they retain many of the characteristics of the original tumour in the patient. Examples of spontaneous models of metastasis using fresh human tumour material and which show metastatic spread in vivo similar to the human disease are shown Table 7b.

## 6 Visualisation of orthotopic and metastatic models

Subcutaneous tumour models are both relatively simple to set up and easy to monitor. Due to the site of tumour growth, the condition of the tumour can be monitored visually and its size measured using callipers, as has been described elsewhere (Stribbling & Ryan, 2022; Tomayko & Reynolds, 1989). However, with the exception of tumours growing in the skin or in the mammary fat pad, more sophisticated methods are required to monitor tumours growing orthotopically. Several non-invasive methods exist to evaluate tumour burden, distribution and response to therapy (Table 3). Of these, bioluminescence imaging (BLI) is the most common (Shen et al., 2020). A disadvantage of BLI is that cells require genetic modification (e.g. to express firefly luciferase) prior to implantation. To detect tumour cells, mice are injected with luciferin. Tumour cells expressing luciferase in the presence of luciferin and oxygen will produce oxyluciferin, CO_2_ and photons (540 nm) in an ATP-dependent reaction. The light produced can be imaged and quantified and can be obtained from the same mouse over a prolonged time period (weeks/months) and so the technique is suitable for following both tumour growth and metastasis. Although BLI has limitations, it is technically simple to perform (requiring an i.p. injection followed by brief anaesthesia while the image is obtained), the data is simple to acquire and the images are straightforward to interpret and understand. Alternative approaches such as fluorescence imaging have similar advantages/disadvantages requiring genetic modification, but are currently less well developed. Techniques such as ultrasound, CT, MRI, PET, and SPECT have all been used to image orthotopic tumour growth, but are limited by technical complexity and sensitivity and/or the need to use radiation or radiotracers which are less suitable for longitudinal studies (de Jong, Essers, & van Weerden, 2014; Lauber et al., 2017; Serkova et al., 2021). For some specialised tumour models such as orthotopic rectal/colorectal cancers, it is possible to use colonoscopy which can provide a detailed visual and quantitative assessment of the primary tumour, but it is not suitable for assessing metastatic spread (Kodani et al., 2013).

## 7 Summary, challenges and future directions

In addition to advancing our understanding of the basic biological processes underlying cancer development, mouse models have played an essential role in the discovery and development of new anti-cancer medicines (Ruggeri et al., 2014), and these new drugs are now contributing to the significant decreases recently seen in cancer mortality rates (Siegel, Miller, Wagle, & Jemal, 2023), at least in some disease settings (Davis et al., 2017; MacEwan et al., 2020; Moreau Bachelard, Coquan, du Rusquec, Paoletti, & Le Tourneau, 2021). Within the context of both biology and drug development it seems likely that orthotopic, metastatic and PDX human tumour models in mice will play an increasing role in improving the success rate of translating preclinical data into improved clinical outcomes (Antonello & Nucera, 2014; Gao et al., 2015a). Crucially, the source material (cell lines, organoids, PDXs) for orthotopic models can be selected to represent the full range of the stages of cancer from early establishment at the primary site to advanced metastases, and each approach has its advantages and disadvantages (Table 8) (Ireson et al., 2019). In addition, orthotopic models capture the key stages of cancer development from initial growth and invasion to growth at metastatic sites. Treatment of advanced disease can encounter intrinsic or acquired drug resistance – a major limitation to current therapies. Importantly, there is a high clinical concordance of drug resistance in PDOX models (Higuchi et al., 2023), reinforcing the clinical relevance of orthotopic models for identifying novel treatment options.

**Table 8:** Advantages and Disadvantages of Different Orthotopic tumour models.

Orthotopic Model	Main Characteristics	Advantages	Disadvantages	Main application
Cell line derived xenograft (CDX) or syngeneic tumour	Xenograft: human tumour cells are injected into their tissue of origin in immune-deficient mice. Syngeneic: murine tumour cells are injected into their tissue of origin in immune-competent mice.	Very straightforward to set up. Many different well-characterised tumour cell lines are very widely available. Intact immune system (syngeneic only).	Cell lines may highly differ from original source tumour. Homogeneous tumour with poorly developed microenvironment. No intact immune system (xenograft).	Confirmation in a disease relevant setting of in vitro and in vivo findings (from subcutaneous models).
Patient-derived xenograft (PDX)	Fresh human tumour tissue from a biopsy or surgery is propagated subcutaneously and transplanted into its tissue of origin in immune-incompetent mice.	The tumour retains the original genetic and histological characteristics, 3D architecture and defined stromal structure. Results are more predictive of the human disease. Fresh tissue enables work with minimally manipulated tumour samples. Tissue obtained pretreatment, on-treatment and post-treatment allows study of various stages of therapy response.	Lengthy time required for tumour establishment and passaging. Low/variable take rate. Human stroma slowly replaced by murine stroma. No intact immune system.	Preclinical trials to predict patient responses in the clinic.
Patient-derived organoid xenograft	Organoids are derived from fresh human tumour tissue from a biopsy or surgery. They are propagated in vitro and transplanted into its tissue of origin in immune-deficient mice.	In vitro propagation is relatively simple. The tumour retains many (but not all) of the original heterogeneous genetic and histological characteristics, 3D architecture and defined stromal structure. Results may be predictive of responses in human disease.	Methods for the derivation and culture of organoids are not standardized. Tumour organoids may not fully capture the heterogeneity present in original tumours. In vitro cell culture propagation may lead to genetic and phenotypic drift. No intact immune system	A potentially more accessible and practicable alternative to PDX
Cell line or organoid derived from genetically engineered mouse (GEM).	Cell lines or organoids are established from tumours isolated from genetically engineered mice.	Straightforward to set up. Tumours arise in defined conditions with known natural history. Tumours are genetically defined and may have conditional activation/inactivation of multiple target genes. Tumours have well-defined stroma and same-species molecular interactions Intact immune system.	Tumours do not have the high level of genetic heterogeneity present in human tumours. Tumours do not generally metastasise. Tumour responses may not be predictive of (more complex) human disease.	Most refined models for investigating tumour biology. Identifying and validating new tumour targets.

Compared with conventional small molecule or antibody-based anti-cancer drugs, targeted drug delivery systems such as nanoparticles are anticipated to have several advantages especially active tumour-targeting and limited normal tissue localisation (Liu et al., 2021). Importantly, orthotopic models, often combined with non-invasive imaging, have provided a key platform to confirm tumour targeting within a relevant tissue setting across a wide range of tumour sites and different targeting platforms (de Paiva et al., 2022; Elumalai, Srinivasan, & Shanmugam, 2024; Jing et al., 2022; Liu et al., 2022; Lu et al., 2013; Wang et al., 2022b). Nonetheless, few targeted drug delivery therapeutics have been approved as anti-cancer therapies (Liu et al., 2021), so it may be important for future studies to investigate whether tumour targeting is retained in more clinically relevant PDOX models in the context of a humanised immune system.

The increasing number of antibody-based therapies and drugs targeting the immune system in development has highlighted the need for the future development both of mouse models that more completely reflect the human immune system (Allen et al., 2019) and for genetically modified mouse models that express functional human versions of mouse proteins that can be targeted by human-specific antibody-directed therapies (Allen et al., 2019).

A limitation of most orthotopic human tumour models is the absence of human cell types in the tumour microenvironment in mice. Although some components of the human immune system can be included by transplantation of precursors into immune-deficient mice, other key stromal cell types such as endothelial cells and fibroblasts, which may be important in therapy response or establishing metastatic niches, are absent and maintaining a fully functioning humanised haematological system (neutrophils, erythrocytes, platelets, lymphatics) is not yet possible, although some significant advances have been made (Shultz, Brehm, Garcia-Martinez, & Greiner, 2012; Theocharides et al., 2016; Zhou et al., 2023b).

Monitoring tumour growth in orthotopic and metastatic tumour models remains a significant challenge (de Jong et al., 2014; Lauber et al., 2017). Whilst bioluminescence imaging is widely used and is generally considered a reliable method to assess tumour burden (Shen et al., 2020), it has technical and biological limitations (Baklaushev et al., 2017; Shen et al., 2020), including the need to genetically modify tumour cells to express luciferase which may affect immune responses (Podetz-Pedersen, Vezys, Somia, Russell, & McIvor, 2014). Alternative optical imaging approaches such as expression of fluorescent proteins are possible (Cool, Breyne, Meyer, De Smedt, & Sanders, 2013), but also require genetic modification and are additionally limited by tissue penetration by light, although this may be addressed by advances in tomography methods and use of near-infrared fluorophores (de Jong et al., 2014) but these approaches have yet to find widespread use.

Non-invasive imaging techniques such as MRI (Ravoori et al., 2019), CT (Myers et al., 2022), and US (Curiel-Garcia, Decker-Farrell, Sastra, & Olive, 2022) allow the detection and monitoring of non-genetically modified orthotopic tumours and may have an advantage over bioluminescence imaging because no genetic manipulation is required and they are more directly translatable to the clinic (de Jong et al., 2014). An important area for further investigation is multimodal imaging of tumours which can combine the benefits of both optical and non-invasive approaches (Haldorsen et al., 2015; Scheepbouwer, Meyer, Burggraaf, Jose, & Molthoff, 2016; Wu et al., 2016).

Positron-emission tomography (PET), most notably ^18^F-fluorodeoxyglucose (FDG) PET is used clinically for the detection of tumours and metastases but is not used widely in preclinical studies of orthotopic tumour growth or response to therapy, primarily because of its lack of spatial resolution (2–3 mm), high cost, and technical complexity. Nonetheless, by combining with optical imaging studies, PET studies can provide important functional information (e.g. metabolism, hypoxia, proliferation) on the effects of treatment on orthotopic tumours which is directly relevant to clinical development (Fu et al., 2016; Fushiki et al., 2013; Haldorsen et al., 2015; Song et al., 2016). In addition, with recent developments of theranostic agents (which couple an ^18^F-labelled diagnostic biomarker and a radiolabelled therapeutic agent) (Bodei, Herrmann, Schöder, Scott, & Lewis, 2022) there is increasing interest in studying ^18^F-labelled ligands such as PSMA and FAPI as PET tracers to determine tumour growth in orthotopic tumour models, again with the potential for direct translation to the clinic (Holzgreve et al., 2021; Kirchner et al., 2021; Zhang et al., 2022).

Established tumour cell lines have been most widely used for orthotopic and metastatic tumour models because they are easy to culture, have been studied in multiple laboratories and importantly are readily available from international tumour cell line banks such as ATCC (www.atcc.org), ECACC (www.ukbrcn.org), and RIKEN (cell.brc.riken.jp). Biobanks of PDX tumours, usually focussing on a particular disease site are being developed (Abdirahman et al., 2020; Bürtin et al., 2021; Damhofer et al., 2015; Elst et al., 2022; Manzella et al., 2020; Matschos et al., 2021; Moy et al., 2022; Tanaka et al., 2022) but the complexities of their growth, maintenance and distribution will likely limit their more widespread use in preclinical studies. However, the recent rapid growth of disease-specific organoid biobanks, including in the major cancer types (Beshiri et al., 2018; Ebisudani et al., 2023; Farin et al., 2023; Shi et al., 2020; Shu et al., 2022) suggests that organoid methodology will play an increasing role in orthotopic tumour models in the future, especially where matched PDX/organoid samples are available (Beshiri et al., 2018; Xu et al., 2023).

A large-scale investigation of established therapies of approximately 1000 subcutaneous PDX tumours representing the major tumour types demonstrated that these models had good predictive value for clinical efficacy (Gao et al., 2015b). Nonetheless, the study also revealed that only a proportion of PDXs for a specific tumour responded to disease-specific therapies, in line with observed interpatient heterogeneity in response observed in clinical studies (Gao et al., 2015b). Consequently, studies using a single or a small number of PDX models to study biology or drug responses may be insufficient to draw firm conclusions, and multiple PDXs of a particular disease type (representing individual patients) may be needed to better estimate clinical efficacy. Importantly, although orthotopic models are thought to be more clinically relevant and therefore potentially more predictive of drug efficacy in patients than subcutaneous tumours (Garber, 2006; Ireson et al., 2019; Killion, Radinsky, & Fidler, 1998b; Villarroel et al., 2009), a comprehensive study to confirm this using patient-derived tumours and up-to-date therapies has yet to be carried out.

Many of the research examples that have been highlighted are from the most common cancer types. However, a significant gap exists in using in vitro and in vivo models, including orthotopic and metastatic models, to study a broad range of rare cancer (defined as incidence of <6 cases per 100,000 people per year) (Gatta et al., 2011) which as a group are often overlooked in terms of research attention. Importantly, when taken together, rare cancers constitute approximately 24% of all prevalent cancers (Gatta et al., 2011) representing an important underserved disease group. However, the ongoing development of disease-specific banks of PDXs and organoids present an important opportunity that should facilitate bringing a broader range of rare tumour types into orthotopic and other mouse models (Li et al., 2022).

Although more technically difficult, more expensive, and placing a greater burden on animal welfare than subcutaneous tumour models, the promise of greater clinical predictivity has driven a growing interest in the use of orthotopic tumour models earlier in the drug development process.

In addition to the insights provided into the biological processes of oncogenesis and metastasis, orthotopic and metastatic models can be utilised at multiple stages of the drug discovery process, each serving specific purposes. In early discovery, orthotopic models can provide stronger validation of potential targets, by implanting cancer cells in the organ of origin to assess the relevance of a specific molecular target in a physiologically relevant context. In later stages (hit-identification and lead optimisation), as drug candidates emerge, orthotopic and metastatic models help assess anti-tumour efficacy and specificity in a more realistic tumour microenvironment. At candidate selection, when comparing several molecules, orthotopic models provide the most relevant physiological setting for choosing potential clinical candidates. As novel agents progress into the clinic, orthotopic models can bridge the gap between preclinical and clinical studies where insights can identify biologically relevant biomarkers and inform the design of early-phase clinical trials (Abou-Elkacem et al., 2013; Higuchi et al., 2018; Kabraji et al., 2023; Patton et al., 2021).

Improved humanised mouse models and increased accessibility to patient-derived tumours and organoids through the establishment of biobanks seems likely to further accelerate this interest. In parallel, further advances in non-invasive imaging techniques that accurately monitor growth of orthoptic and metastatic disease in mice offer a more direct translation of preclinical findings into the clinic. In conclusion, orthoptic tumour models now appear poised to take their place alongside subcutaneous tumour models as an essential component of the preclinical evaluation of new anti-cancer therapies.

## CRediT authorship contribution statement

**Stephen M. Stribbling:** Writing – review & editing, Writing – original draft, Conceptualization. **Callum Beach:** Writing – review & editing. **Anderson J. Ryan:** Writing – review & editing, Writing – original draft, Funding acquisition, Conceptualization.

## Declaration of competing interest

The Authors report no conflicts of interest.

The Authors have no affiliations with or involvement with any organization or entity with any financial or commercial interest (such as honoraria; educational grants; participation in speakers' bureaus; membership, employment, consultancies, stock ownership, or other equity interest; and expert testimony or patent-licensing arrangements) in the subject matter or materials discussed in this manuscript.
